# Systematic identification and characterization of virus lncRNAs suggests extensive structural mimicry of host lncRNAs

**DOI:** 10.1093/bib/bbaf640

**Published:** 2025-12-01

**Authors:** Ping Fu, Zena Cai, Ruina You, Lei Deng, Zhaoyong Li, Zhichao Miao, Yousong Peng

**Affiliations:** Bioinformatics Center, College of Biology, Hunan Provincial Key Laboratory of Medical Virology, Hunan Research Center of the Basic Discipline for Cell Signaling, Hunan University, No. 27 Tianma Road, Yuelu District, Changsha, 410082, China; State Key Laboratory of Chemo/Biosensing and Chemometrics, Longping Agricultural College, Hunan University, No. 27 Tianma Road, Yuelu District, Changsha 410082, China; Bioinformatics Center, College of Biology, Hunan Provincial Key Laboratory of Medical Virology, Hunan Research Center of the Basic Discipline for Cell Signaling, Hunan University, No. 27 Tianma Road, Yuelu District, Changsha, 410082, China; Department of Medical Genetics, Hunan Provincial Maternal and Child Health Care Hospital, No. 53 Xiangchun Road, Kaifu District, Changsha 410028, China; Bioinformatics Center, College of Biology, Hunan Provincial Key Laboratory of Medical Virology, Hunan Research Center of the Basic Discipline for Cell Signaling, Hunan University, No. 27 Tianma Road, Yuelu District, Changsha, 410082, China; Bioinformatics Center, College of Biology, Hunan Provincial Key Laboratory of Medical Virology, Hunan Research Center of the Basic Discipline for Cell Signaling, Hunan University, No. 27 Tianma Road, Yuelu District, Changsha, 410082, China; Bioinformatics Center, College of Biology, Hunan Provincial Key Laboratory of Medical Virology, Hunan Research Center of the Basic Discipline for Cell Signaling, Hunan University, No. 27 Tianma Road, Yuelu District, Changsha, 410082, China; GMU-GIBH Joint School of Life Sciences, The Guangdong-Hong Kong-Macau Joint Laboratory for Cell Fate Regulation and Diseases, Guangzhou National Laboratory, Guangzhou Medical University, No. 190 Kaiyuan Road, Guangzhou Science Park, Luogang District, Guangzhou 510530, China; Bioinformatics Center, College of Biology, Hunan Provincial Key Laboratory of Medical Virology, Hunan Research Center of the Basic Discipline for Cell Signaling, Hunan University, No. 27 Tianma Road, Yuelu District, Changsha, 410082, China

**Keywords:** bioinformatics, virus, long noncoding RNAs, third-generation sequencing, host mimicry

## Abstract

Virus long noncoding RNAs (vlncRNAs) play crucial roles in viral infections, yet their identification and characterization remain limited. This study identified 5,053 novel vlncRNAs across 25 viral species using third-generation sequencing, with two from Influenza A virus and Vesicular stomatitis virus validated by RT-qPCR. Most vlncRNAs originated from dsDNA viruses. Only ~1% of vlncRNAs have annotated RNA families, suggesting many novel RNA structures. Interestingly, a total of 772 vlncRNAs from 15 human viruses structurally mimicked human lncRNAs (hlncRNAs), while only seven vlncRNAs shared sequence similarities with hlncRNAs. These vlncRNA and hlncRNAs bound to similar miRNAs, potentially acting as miRNA sponges to promote essential life processes. Splicing analysis showed vlncRNAs had a prevalence of alternative first exon. Finally, we developed vlncRNAbase (http://computationalbiology.cn/vlncRNAbase/#/) to store and organize the newly identified and known vlncRNAs. Overall, the study provides a valuable resource for further investigation into vlncRNAs and deepens our understanding of the diversity, structure, and function of the molecule.

## Introduction

The long noncoding RNA (lncRNA) is a prominent class of noncoding transcripts that have longer than 200 nt and lack the protein-coding ability [1]. The majority of lncRNAs are transcribed by the RNA polymerase II, with typical 5′ capping and 3′ polyadenylated tailing [2]. The lncRNAs have been widely found in humans, animals, plants, fungi and prokaryotes [3]. They have diverse functions, including modulating gene expression [4], being involved in chromatin structure [5] and contributing to cell differentiation [6]. The lncRNAs play an important role in multiple human diseases, including cancer [7–9]. For example, the lncRNA PVT1 was reported to promote ovarian cancer progression by silencing miR-214, a miRNA commonly involved in carcinogenesis [7]. Additionally, the lncRNA PNUTS was involved in breast cancer metastasis through its impact on the epithelial-mesenchymal transition process [8]. Thus, they have been considered crucial components of the complex regulatory network involved in disease development [9].

The rapid development of high-throughput sequencing technology has greatly facilitated the identification and characterization of lncRNAs [10, 11]. Numerous lncRNAs have been identified based on the next-generation-sequencing (NGS) technology, and several lncRNA databases have been built [12–14]. For example, the lncRNAfunc database contains 15,900 human lncRNAs that are involved in 33 cancer types [12] and the JustRNA database covers 1,088,565 lncRNAs identified from 80 plant species [13]. However, the limitations of NGS, such as short read lengths and potential PCR amplification errors or bias pose great challenges for accurately identifying and quantifying lncRNAs [15, 16]. In contrast, the third-generation-sequencing (TGS) is considered as a more suitable approach for studying lncRNAs due to its ability to generate long reads and single-molecules [17]. For instance, Wan *et al.* discovered ~28,000 lncRNAs in the mouse retina using the full-length isoform sequencing, with 3.4% of them belonging to intergenic lncRNAs (lincRNAs) [18]. Guan *et al.* identified 16,495 lncRNAs from 15,512 loci across 19 chicken tissues using Oxford Nanopore long-read sequencing, uncovering additional novel loci (e.g. lncRNA loci) and resolving complex transcripts (e.g. the longest transcript for the TTN locus), which is often overlooked by NGS [19]. Recovering full-length lncRNA sequences facilitates a comprehensive understanding of their structure and functionality [18, 19].

Recent evidence has highlighted the ability of viruses to encode lncRNAs despite their small genomes [20–24]. Multiple viruses have been reported to encode lncRNAs, such as Kaposi’s Sarcoma-Associated Herpesvirus (KSHV), Human Cytomegalovirus (HCMV), and human immunodeficiency virus (HIV). The virus lncRNAs (vlncRNAs) have been reported to play important roles throughout the viral life cycle [21]. For example, the vlncRNA PAN encoded by KSHV was found to activate viral replication and regulate viral gene expression [21]. The vlncRNA 2.7 encoded by the HCMV was reported to interact with complex I and prevents the translocation of GRIM-19, a gene associated with retinoid interferon-induced mortality, thereby maintaining the mitochondrial membrane potential and ultimately favoring viral ATP production [22, 23]. Additionally, the vlncRNA ASP of HIV can recruit Polycomb Repressor Complex 2 to the 5′ LTR of HIV-1 to establish the latency of HIV-1 [24]. These findings highlight the importance of vlncRNAs in regulating host cells during viral infections. Nevertheless, a comprehensive resource for vlncRNAs is still lacking.

The structure and function of vlncRNAs remain largely unexplored [25]. LncRNAs can act as molecular scaffolds, miRNA sponges, or regulators of gene expression by interacting with proteins, DNA, or RNA [12]. It is difficult to resolve the structure of lncRNAs due to the intrinsic flexibility of the molecule [25]. Computational methods have become indispensable for studying the structure and function of lncRNAs [26–29], such as molecular docking, molecular dynamics simulations, umbrella sampling for RNA-ligand interaction analysis, and RNAdistance for predicting and comparing RNA secondary structures [30]. In this study, we systematically identified vlncRNAs for more than 20 viral species by mining viral infection-related TGS data and further characterized their structure and function. A database named vlncRNAbase was further built to store and organize these newly identified and known vlncRNAs. It would greatly facilitate further research on vlncRNAs in the field.

## Materials and methods

### The workflow of identifying vlncRNAs

**Figure 1:**
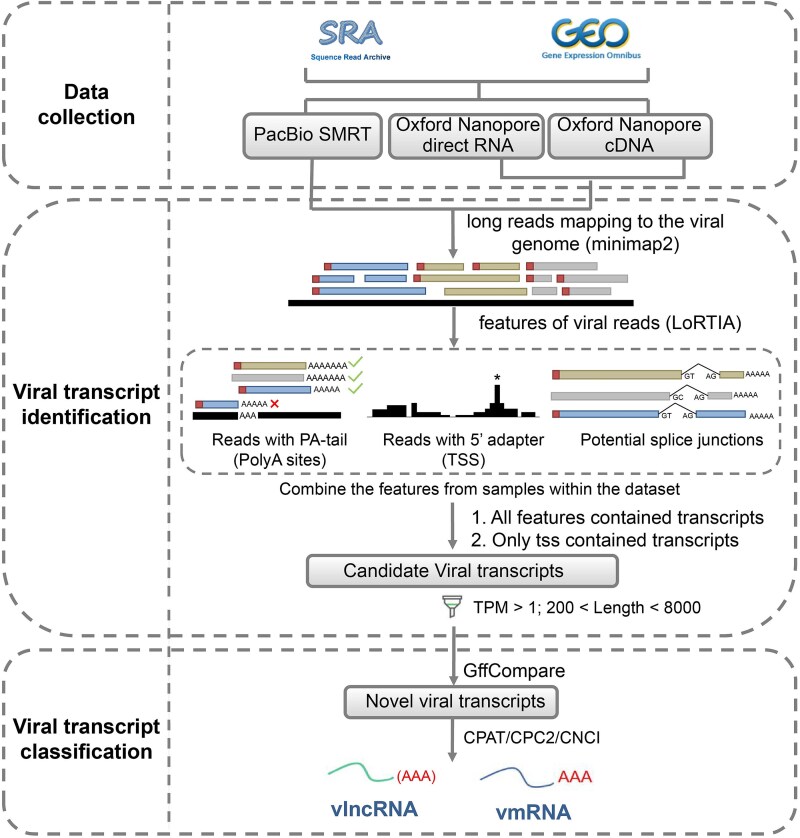
Workflow of identifying vlncRNAs. It contains three modules including data collection, viral transcript identification and viral transcript classification.

The workflow for identifying vlncRNAs can be separated into three modules (Fig. 1). The first module is Data collection, during which the viral infection-related TGS data generated by both Oxford Nanopore and PacBio SMRT technologies were obtained from NCBI GEO and SRA databases. The second module is Viral transcript identification, which includes identification of viral reads through three features: transcription start site (TSS), transcription end site (TES), and potential splice sites [31], and filtering of viral transcripts by abundance and length. The third module is Viral transcript classification, which includes the identification of novel transcripts and the classification of viral transcripts into vmRNA or vlncRNA based on their coding potential.

### Data collection

The viral infection-related TGS datasets were collected from the NCBI SRA and GEO databases in June 2023. The keywords of “pacbio” AND “virus” or “nanopore” AND “virus” were used to query for viral infection-related TGS datasets from the two databases mentioned above. Then, only the datasets with the Library Source of “TRANSCRIPTOMIC” and Assay Type of “RNA-seq” were kept. Subsequently, the remaining datasets were inspected manually. A total of 36 TGS datasets, including 870 samples, were collected and used in the analysis, as provided in Table S1. The viral genomes used in these datasets and their related annotation files were manually collected from the NCBI GenBank database.

### Viral transcript identification

Firstly, viral transcripts were identified by mapping reads to viral genomes using minimap2 (version 2.17) [32]. The parameter settings differed depending on the sequencing type of the TGS data according to the official recommendation of minimap2: the parameter settings of “-ax splice:hq -uf”, “-ax splice -uf -k 14”, and “-ax splice -k 14” were used for PacBio SMRT, Oxford Nanopore direct RNA, and Oxford Nanopore cDNA, respectively.

Second, to detect and annotate viral transcripts, we used LoRTIA (version 0.9.9) [33] to analyze the mapped reads, using the following steps according to Fülöp’s study [31]. For quality control, we applied “--five_score 16 --three_score 18 --check_in_soft 15” for PacBio sequencing and “--five_score 16 --three_score 16 --check_in_soft 15” for Nanopore sequencing. [1] The adapter and polyA sequences at the ends of the reads were checked to identify TSS and TES, respectively. [2] A Poisson distribution test was applied to the putative TSSs and TESs to eliminate random start and end sites, with Bonferroni correction for significance. [3] Potential splice junctions were identified, and those with common splice signals (GT/AG, GC/AG, and AT/AC) were retained. Transcript features including TSS, TES and splice junctions were kept if they were detected in at least two reads and their read frequency exceeds 1‰ of all mapped reads to the viral genome. To improve accurate annotations, we combined features identified from different samples within the same dataset and filtered out viral transcripts supported by less than five reads. Finally, based on the accepted TSSs, TESs, and splice junctions, the sequences of these regions were combined to form viral transcripts. Since not all viral transcripts contain polyA sequences [34], we also retained transcripts containing only TSS and determined their TES by selecting the longest read among mapped reads that had the same TSS. The transcripts with both similar TSS and TES (distance <20 nt) were regarded as the same viral transcript, considering potential inaccuracies in the identification of TSS and TES. Then, the retained viral transcripts were filtered by abundance and length. More than 90% of the vlncRNAs exceeded one TPM (peak 3.69 TPM; Fig. S1A), and 98.2% ranged from 200 to 8,000 nt (median 445 nt; Fig. S1B). Only transcripts with abundance >1 TPM (transcript per million) in at least one sample and length < 8,000 nt were kept for further analysis.

### Viral transcript classification

Firstly, novel viral transcripts were identified using GffCompare (version 0.11.2) [35]. Only transcripts with class_code of “j”, “k”, “m”, “n”, which represent novel isoforms of known genes, and those of “i”, “x”, “u”, “y”, “o”, which potentially represent novel genes or transcriptional units [36], were taken as novel transcripts and were provided in Table S2.

Then, three software tools including CPAT (version 3.0.4, parameter settings: “--antisense --top-orf = 5”) [37], CPC2 (version 0.1, default parameter settings) [38], CNCI (version 2, parameter settings: “-m ve”) [39], were used to predict the coding potential of novel viral transcripts. If a transcript was predicted to be noncoding by at least two tools, or if it was predicted to be noncoding by one tool when there was only one prediction, it was considered as a lncRNA; otherwise, it was considered an mRNA.

### RNA family identification of vlncRNAs

To identify RNA families in vlncRNAs, firstly, the Rfam database (v14.9) [40], a database of RNA families, was downloaded from https://ftp.ebi.ac.uk/pub/databases/Rfam/14.9/. Then, the cmscan program from Infernal (v1.1.2) [41] with parameter settings of “--rfam --nohmmonly --fmt 2 --cut_ga” was used to identify RNA families in the Rfam database based on vlncRNA sequences. All RNA family annotation results for vlncRNAs were summarized in Table S3.

### Analysis of host RNA mimicry of vlncRNAs

Since most TGS data were related to human viruses, we focused on the human RNA mimicry of vlncRNAs. Firstly, sequences of human lncRNAs were collected from Ensembl (release-109, https://ftp.ensembl.org/pub/release-109/fasta/homo_sapiens/ncrna/). Then, the MMseqs2 (version 6.f5a1c) [42] with the easy-cluster mode and with parameter setting of “--min-seq-id 1 --cov-mode 0” was used to remove sequence redundancy at 100% level for both human lncRNAs and novel vlncRNAs. The sequence similarity between human lncRNAs and novel vlncRNAs was analyzed using BLASTN (version 2.9.0+, with the “-word_size 11” parameter) [43]. The vlncRNA was considered to be similar to human lncRNAs if the e-value was smaller than 1E-5 in the blast results. The secondary structure of human lncRNAs was predicted using LinearFold (version 1.0). Subsequently, the RNAdistance (version 2.6.4) [30] with default parameters from the ViennaRNA package was employed to calculate the distance, i.e. the fscore (the lower fscore indicates higher structure similarity), between a pair of human lncRNAs and vlncRNAs. The fscores between all pairs of human lncRNAs and vlncRNAs were ranked in an increasing order. The 5% percentage of these fscores (329) was taken as the threshold, which is much stricter than those used by previous studies [44–46] such as the lower quartile of fscores used by Leung [45]. A pair of human lncRNA and vlncRNAs was considered similar if the distance between them was smaller than the threshold. RNA secondary structures were visualized by forna in the ViennaRNA package [30].

For negative control, a null distribution of structural similarity scores was generated. Specifically, 100 human lncRNAs and 100 viral lncRNAs were randomly selected. For each vlncRNA, 100 sequence-shuffled vlncRNAs were created, yielding 10,000 shuffled sequences. Pairwise similarities were calculated using RNAdistance, resulting in 1,000,000 fscores between hlncRNAs and shuffled vlncRNAs.

### Predicting interactions between human miRNAs and virus-like hlncRNAs as well as human-mimicry vlncRNAs

A total of 1,917 human miRNAs were obtained from miRBase (v22, https://www.mirbase.org/) [47]. The interactions between human miRNAs and virus-like hlncRNAs as well as human-mimicry vlncRNAs were predicted using miRanda (version 3.3a) [48]. To minimize the false-positive rate of the method [49], a strict criterion was used: only a seed region complementarity score (sc) >200, and energy <−40 kcal/mol were used for further analysis.

### Functional analysis of human lncRNAs and human miRNAs

The Gene Ontology (GO) and Kyoto Encyclopedia of Genes and Genomes (KEGG) enrichment analysis was conducted to reveal the functions of hlncRNAs and miRNAs using the *enrichGO()* and *enrichKEGG()* functions in the clusterProfiler package (version 4.8.3) [50]. GO terms and KEGG pathways with FDR adjusted *P* < .05 were considered significantly enriched.

### Analysis of the alternative splicing of vlncRNAs

To investigate the alternative splicing (AS) of vlncRNAs and vmRNAs, SUPPA (version 2.3) [51] with parameters settings of “generateEvents -f ioe -e SE SS MX RI FL” was used to identify the type of splicing events.

### Experimental validation of vlncRNAs

Cells: the Madin-Darby canine kidney (MDCK) cell line, HEK293T cells, and Vero cells were purchased from ATCC. The Huh7 cell line was kindly shared by Haizhen Zhu (Hunan University, Changsha, China). All cells were propagated in Dulbecco’s modified Eagle medium (DMEM) supplemented with 10% Fetal Bovine Serum (FBS), and 1% penicillin–streptomycin. Cells were maintained in a humidified atmosphere of 5% CO_2_ at 37 °C.

Viruses: Indiana vesiculovirus (VSV) was kindly shared by Haizhen Zhu (Hunan University, Changsha, China). The strain A/California/07/2009 of Influenza A virus (IAV) and VSV were propagated and amplified at MOI of 0.001 in chicken embryos and HEK293T cells, respectively. Supernatants were harvested and clarified by centrifugation, then stored at −80 °C. Viral titers were determined by plaque assay in Vero cells.

Real-time PCR assay: then, utilizing the aforementioned amplified viruses with MOI = 0.5, Huh7 cells were infected with VSV and cultured for an additional 9 h, while MDCK cells were infected with IAV and cultured for an additional 24 h. Subsequently, the total cellular RNA was isolated using TRIzol Reagent (Invitrogen). After the RQ1 DNase (Promega) treatment, the extracted RNA was used for the synthesis of the first strand of cDNA with the Superscript III first-strand Synthesis System (Invitrogen) as described previously [34, 52]. After the reverse transcription (RT), the generated template cDNAs were utilized for real-time quantitative PCR (qPCR) analysis. To reduce nonspecific amplification, qPCR primers were designed to strategically target alternative splice sites in vlncRNAs. The primers used for RT-qPCR are listed in Table S4. Expression levels of vlncRNAs were normalized by mRNA of the housekeeping gene GAPDH. The relative expression levels of vlncRNAs were calculated using the method of 2-^△△^ct.

### Database implementation

The front-end and back-end separation frameworks were used to implement vlncRNAbase which was described in our previous study [53]. The JavaScript Object Notation format was employed to implement the communication between the client-side and server-side layers. The data were stored in a MySQL (version 5.5.62) database [53].

### Statistical analysis

All statistical analyses were conducted in Python (version 3.8) and R (version 4.3.1). The Wilcoxon rank-sum test was conducted using the function of *wilcox.test()* in R. A *P* < .05 was considered statistically significant.

## Results

### Identification and validation of novel vlncRNAs from TGS data

**Figure 2:**
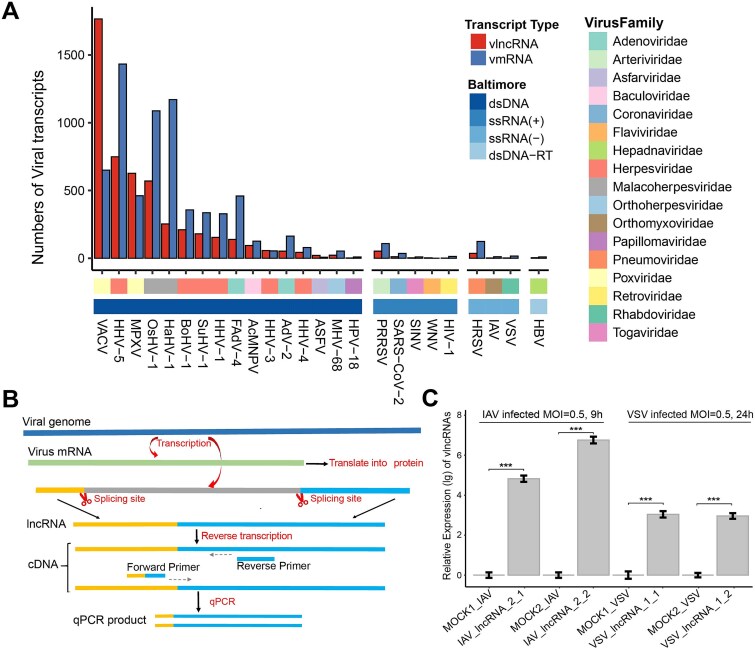
Identification and characterization of novel vlncRNAs and vmRNAs. (A) The number of novel vlncRNAs (red) and vmRNAs (blue) in 25 virus species. The first bar below the x-axis represents the Baltimore group to which the virus belongs, while the second bar represents the virus family. For clarity, the abbreviation of virus names was used. Supplementary Table S5 displays the full names of these viruses. dsDNA, double-stranded DNA virus; ssRNA (+), positive-sense single-stranded RNA virus; ssRNA (−), negative-sense single-stranded RNA virus; dsDNA-RT, double-stranded DNA reverse transcribing virus. (B) Diagram of designing primers specific for detecting vlncRNAs by RT-qPCR. (C) The relative expression of IAV_lncRNA_2 and VSV_lncRNA_1 by RT-qPCR following amplification with two pairs of primers. For clarity, the expression values were log_10_- transformed. ^***^: *P* < .001.

A total of 5,053 novel vlncRNAs and 7,101 novel vmRNAs were identified in 25 virus species from 17 virus families that belonged to four Baltimore groups based on computational analysis of 870 viral infection-related samples of TGS data (Materials and Methods) (Fig. 2A and Fig. S2). Most novel vlncRNAs were identified from double-stranded DNA (dsDNA) viruses (97.94%), then the ssRNA (+) (1.39%) and ssRNA (−) (0.79%). The Vaccinia virus (VACV) of the *Poxviridae* family had the highest number of novel vlncRNAs (1766), followed by Human betaherpesvirus 5 (HHV-5) (749) and Monkeypox virus (MPXV) (627). Similarly, most novel vmRNAs were also identified from dsDNA viruses (95.40%). The HHV-5 of the *Herpesviridae* family, Haliotid herpesvirus 1 (HaHV-1), and Ostreid herpesvirus 1 (OsHV-1) from the *Malacoherpesviridae* family had the largest number of novel vmRNAs, with 1,433, 1,171, and 1,088, respectively. The number of novel vmRNAs exceeded that of novel vlncRNAs for all viruses except VACV, MPXV, Human alphaherpesvirus 3 (HHV-3), African swine fever virus (ASFV), and West Nile virus (WNV).

To validate the vlncRNAs, two vlncRNAs of IAV, IAV_lncRNA_1 from the M1 segment and IAV_lncRNA_2 from the PB1 segment, and two vlncRNAs of VSV, VSV_lncRNA_1 from the gp2 gene and VSV_lncRNA_2 from the gp1 gene, were selected for validation by RT-qPCR (Materials and Methods). To ensure that the primers specifically amplify the target fragments, we designed two pairs of primers targeting the alternative splice sites of each vlncRNA (Fig. 2B, Fig. S3 and Table S4). Subsequently, RT-qPCR was used to quantify the relative expression levels of these vlncRNAs. Two vlncRNAs, i.e. the IAV_lncRNA_2 and VSV_lncRNA_1, exhibited high expression levels at 9 hours post-infection (hpi) and 24 hpi, respectively. The former showed relative fold changes of 4.82 and 6.76 for two pairs of primers after log_10_ transformation, while the latter had 3.04 and 2.96 for two pairs of primers after log_10_ transformation (Fig. 2C). In contrast, almost no expression was detected in the mock-infected group.

### Length, GC content, and abundance of vlncRNAs

**Figure 3:**
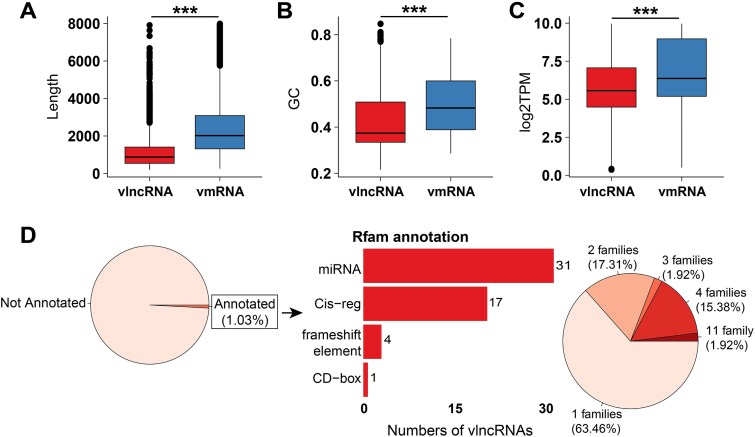
Sequence and structure characteristics of novel vlncRNAs. (A–C) refer to the length, GC content and expression distribution of novel vmRNAs and vlncRNAs, respectively. For clarity, the expression of RNAs were log_2_-transformed. (D) The pie chart at left refers to the portion of the vlncRNAs annotated in Rfam. The bar plot in the middle shows the number of vlncRNAs that were classified into four RNA types based on the Rfam. The pie chart at right shows the proportion of vlncRNAs that were annotated to one or multiple RNA families.

Then, the length, GC content, and abundance of vlncRNAs and vmRNAs were analyzed and compared (Figs 3A–C). The vlncRNAs were found to have significantly shorter length, lower GC content, and lower expression level compared to those of vmRNAs. Specifically, in terms of length, vmRNAs were twice as long as vlncRNAs, with the former having a median of 2,020 nt and the latter having a median of 876 nt. Concerning the GC content, vmRNAs had a median of 48.27%, while vlncRNAs had a median of 37.40%. Regarding the abundance, vmRNAs and vlncRNAs had median log_2_TPM values of 6.35 and 5.54, respectively.

### Analysis of the structural characteristics of novel vlncRNAs

Then, RNA families of the vlncRNAs were annotated to further characterize their structure based on the Rfam database (Materials and Methods). Only 52 vlncRNAs (1.03% of all vlncRNAs) were annotated and predicted to contain 1–11 RNA families. Among them, 63.46% had only one annotated RNA family (Fig. 3D). A total of 33 RNA families were identified in vlncRNAs and they were further grouped into four RNA types including “miRNA” i.e. defined as small noncoding and single-stranded RNAs involved in the gene expression regulation, “Cis-reg” i.e. defined as functional regulatory elements to regulate the transcription of neighboring genes, “frameshift element” i.e. defined as a sequences that facilitate ribosomal frameshifting during translation, and “CD-box” that was defined as snoRNA with C (UGAUGA) and D (CUGA) box motifs. “miRNA” was the most frequently annotated RNA type, with 31 vlncRNAs; “Cis-reg” was the secondly annotated RNA type, containing 17 vlncRNAs. The other two RNA types each contained less than five vlncRNAs. Similarly, RNA families of the vmRNAs were annotated and grouped into four types: “Cis-reg”, “miRNA”, “frameshift element”, and “Epstein-Barr virus stable intronic sequence RNA 2”, with “Cis-reg” being the most frequent (74.69%), followed by “miRNA” (17.50%); the other two types were less common (Fig. S4).

### Extensive structural mimicry of human lncRNAs by vlncRNAs

**Figure 4:**
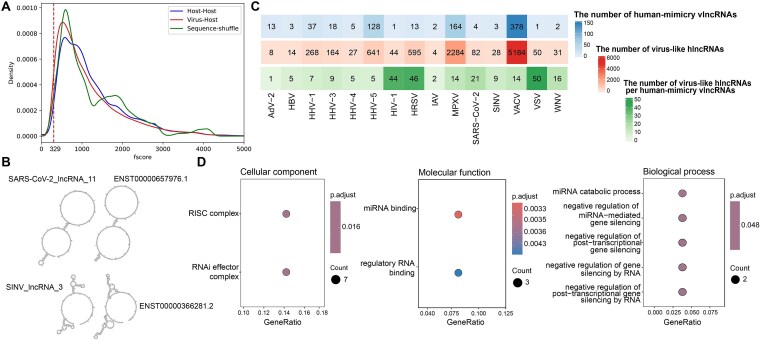
Analysis of hlncRNAs mimicry of vlncRNAs. (A) The density distribution of fscores between vlncRNAs and hlncRNAs (red), among hlncRNAs (blue) as well as sequence-shuffled vlncRNA and hlncRNA (green). The dashed line represents the 5% threshold for the similarity between vlncRNAs and hlncRNAs. (B) An example of the secondary structure of SARS-CoV-2_lncRNA_11 compared to ENST00000657976.1 and SINV_lncRNA_3 compared to ENST00000366281.2. (C) The number of human-mimicry vlncRNAs and virus-like hlncRNAs across 15 human viruses. The first row (blue) displays the number of human-mimicry vlncRNAs, the second row (red) presents the number of virus-like hlncRNAs, and the third row (green) displays the number of virus-like hlncRNAs per human-mimicry vlncRNAs. (D) Enriched GO functions (cellular component, molecular function, and biological process) for virus-like hlncRNAs that were structurally similar to vlncRNAs of at least eight viruses.

Previous studies have shown that viral RNAs such as miRNAs, may mimic the host RNAs to suppress innate immunity and hijack host machinery for their survival [54]. Thus, the sequence and structure similarity between vlncRNAs and human lncRNAs (hlncRNAs) was analyzed, as most viruses analyzed were human-infecting. Only 0.14% of vlncRNAs had sequence similarity with hlncRNAs (Materials and Methods), suggesting little sequence similarity between vlncRNAs and hlncRNAs. Then, the similarity of secondary structures between all pairs of vlncRNAs and hlncRNAs was analyzed (Materials and Methods) (Fig. 4A). A median fscore (smaller fscore indicates higher similarity) of 906 was observed between vlncRNAs and hlncRNAs. For comparison, we randomly selected 1,000 hlncRNAs and calculated the structure similarity among them. A median fscore of 1,023 was obtained for hlncRNAs, which was significantly larger than that between vlncRNAs and hlncRNAs (*P* < 2.2E-16 in the Wilcoxon rank-sum test). In addition, a negative control was performed by using sequence-shuffled vlncRNAs. The shuffled vlncRNA-hlncRNA mimic pairs showed a median fscore of 959, which was also significantly larger than the observed vlncRNA-hlncRNA similarity (*P* < 2.2E-16 in the Wilcoxon rank-sum test). This suggests that vlncRNAs had greater structural similarity to hlncRNAs than the hlncRNA do with each other, and the structural similarity of vlncRNAs and hlncRNAs were not a result of sequence or nucleotide composition similarity.

The top 5% pairs between vlncRNAs and hlncRNAs were considered as structural similar, which involved 772 vlncRNAs (defined as human-mimicry vlncRNAs) from 15 human viruses and 9,393 hlncRNAs (defined as virus-like hlncRNAs). For example, the secondary structure of SARS-CoV-2_lncRNA_11 contained three loops connected by two stems that were highly similar to the secondary structure of ENST00000657976.1, which is derived from LINC02516 and is an intergenic lncRNA located on chromosome 4 in the hg38 genome assembly. Likewise, the secondary structure of SINV_lncRNA_3 was similar to that of ENST00000366281.2, which is transcribed from the SLC44A4 gene and is located on chromosome 6 in hg38 genome assembly (Fig. 4B).

Human viruses exhibited a range of 1–378 human-mimicry vlncRNAs, with a median of 5. VACV had the highest number of human-mimicry vlncRNAs (378). On the other hand, the number of virus-like hlncRNAs also varied across viruses, ranging from 8 to 5,184. VACV had the largest number of virus-like hlncRNAs (5,184), followed by MPXV (2,284) and HHV-5 (641). We further calculated the number of virus-like hlncRNAs per human-mimicry vlncRNAs (Fig. 4C). Interestingly, the VSV had the highest number of virus-like hlncRNAs per human-mimicry vlncRNAs [50], followed by Human Respiratory Syncytial Virus (HRSV) [46] and Human alphaherpesvirus 1 (HHV-1) [44].

Then, the functions of vlncRNAs were investigated by analyzing the functions of virus-like hlncRNAs, as similar structures may suggest similar functions. A total of 1,466 virus-like hlncRNAs were found to be structurally similar to vlncRNAs in at least half of viruses analyzed (8 out of 15). These virus-like hlncRNAs were enriched in a limited number of GO terms (Fig. 4D) and showed no significant enrichment in the KEGG pathway. Specifically, the enriched cellular components included “RISC complexes” and “RNAi effector complexes”; the enriched molecular functions included “miRNA binding” and “regulatory RNA binding”; the enriched biological processes were primarily associated with the negative regulation of gene silencing, such as “negative regulation of miRNA-mediated gene silencing” and “negative regulation of post-transcriptional gene silencing”.

### Human-mimicry vlncRNAs and virus-like hlncRNAs binding to similar miRNAs

**Figure 5:**
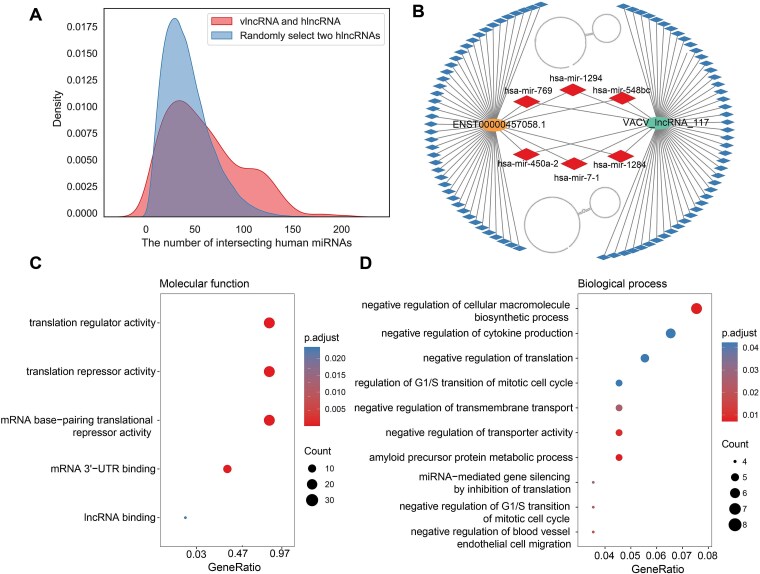
Analysis of miRNAs interacting with both human-mimicry vlncRNAs and virus-like hlncRNAs. (A) Density distribution of intersecting human miRNAs between human-mimicry vlncRNAs and their corresponding virus-like hlncRNAs (red), compared to that of randomly selected human lncRNA pairs (blue). (B) An example of a pair of human-mimicry vlncRNAs and virus-like hlncRNAs binding to similar miRNAs. The secondary structures of the hlncRNA (top) and vlncRNA (bottom) are shown. miRNAs are represented by diamonds, with red ones indicating miRNA that interact with both hlncRNA and vlncRNA. (C and D) Enriched molecular functions and biological process for human miRNAs that interact simultaneously with virus-like hlncRNAs and human-mimicry vlncRNAs.

The lncRNAs can bind to miRNAs to take part in post-transcriptional processes [7]. Our results showed that the virus-like hlncRNAs were mainly involved in miRNA-related functions (Fig. 4D). Thus, we further investigated whether the human-mimicry vlncRNAs and their corresponding virus-like hlncRNAs bind to similar miRNAs. The analysis showed that nearly all human-mimicry vlncRNAs (768/772) bound to similar miRNAs as their corresponding virus-like hlncRNAs. The number of shared miRNAs between human-mimicry vlncRNAs and virus-like hlncRNAs ranged from 0 to 205, with a median of 52 (colored in red in Fig. 5A). For instance, the VACV_lncRNA_117 and its corresponding virus-like hlncRNA ENST00000457058.1 had highly similar structures (Fig. 5B). They interacted with 48 and 51 human miRNAs, respectively. Of these, six miRNAs bound to both lncRNAs (colored in red in Fig. 5B). For comparison, we also analyzed the number of shared miRNAs between human lncRNAs (colored in light blue in Fig. 5A) and found that the human lncRNAs shared a median of 38 miRNAs which was significantly smaller than that between human-mimicry vlncRNAs and virus-like hlncRNAs (*P* < 2.2E-16 in the Wilcoxon rank-sum test).

The functions of 505 human miRNAs that interacted simultaneously with virus-like hlncRNAs and human-mimicry vlncRNAs were investigated by the GO and KEGG pathway enrichment analysis. A limited number of functions were enriched. The enriched molecular functions were mainly related to translation regulation, such as “translation regulator activity” and “mRNA base-pairing translational repressor activity” (Fig. 5C). The enriched biological processes were mainly related to negative regulation of multiple essential life processes such as cellular macromolecule biosynthesis, cytokine production, translation, transmembrane transport and regulation of cell cycle such as G1/S transition of the mitotic cell cycle (Fig. 5D). The enriched cellular components included the RISC complex and RNAi effector complex pathways, similar to those for virus-like hlncRNAs (Fig. S5A). The enriched KEGG pathway included the “microRNA in cancer” pathway (Fig. S5B).

### Expression correlation between human-mimicry vlncRNAs and virus-like hlncRNAs

Then, the relationship between the expression of human-mimicry vlncRNAs and corresponding virus-like hlncRNAs was investigated. The analysis revealed a Pearson correlation coefficient (PCC) of 0.20 (*P* < .001) (Fig. S6A), whereas randomly selected nonmimicry hlncRNAs had a PCC of 0.01 (*P* = 0.53 ) (Fig. S6B), indicating a weak but statistically significant positive correlation between the expression level of these two RNA types.

### Generation dynamics of vlncRNAs

**Figure 6:**
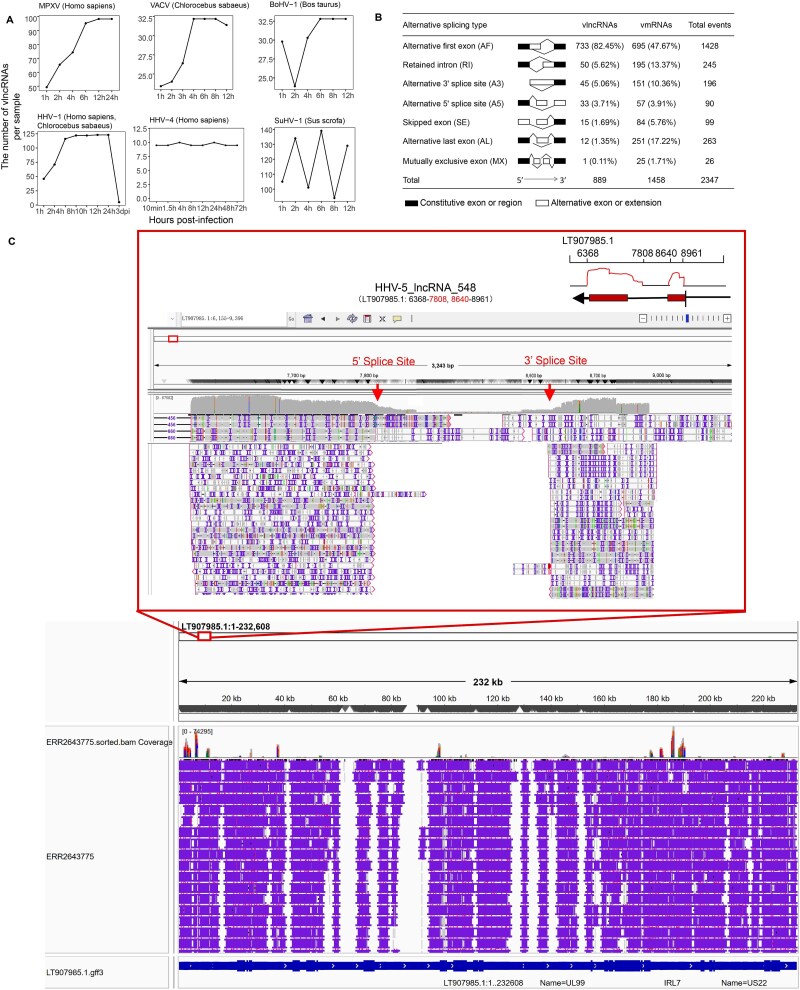
Analysis of vlncRNA generation dynamics and the biogenesis mechanism. (A) The number of vlncRNAs per sample detected at different hours post-infection in Monkeypox virus (MPXV), Vaccinia virus (VACV), Bovine alphaherpesvirus 1 (BoHV-1), Human alphaherpesvirus 1 (HHV-1), Human gammaherpesvirus 4 (HHV-4), Suid alphaherpesvirus 1 (SuHV-1). All time points from each dataset were included. (B) Analysis of the alternative splicing pattern of vlncRNAs. (C) Example of an alternative first exon (AF) splicing event in the viral lncRNA HHV-5_lncRNA_548.

To explore the expression dynamics of vlncRNA during viral infections, we analyzed publicly available RNA-seq time-course datasets from 13 viruses, each sampled at multiple post-infection time points. Notably, only HHV-1 was represented by datasets from multiple hosts (*Homo sapiens* and *Chlorocebus sabaeus*); all other viruses were analyzed within a single host. For most of these viruses, the number of vlncRNAs per sample increased as viral infections progressed (Fig. 6A and Fig. S7). For example, in MPXV, the number of vlncRNAs per sample increased from 49 at 1 hpi to 74 at 4 hpi, and further to 98 at 12 hpi. Similarly, in VACV, the number of vlncRNAs per sample increased from 23 at 1 hpi to 26 at 3 hpi, and further to 32 at 8 hpi (Fig. 6A). Some virus such as HHV-4 showed a stable ability of generating vlncRNAs during the infection process, while some viruses such as HHV-1 and SuHV-1 showed complex vlncRNA expression patterns.

### Analysis of the alternative splicing pattern of vlncRNAs

To investigate the biogenesis mechanism of vlncRNAs, we analyzed seven common AS patterns of vlncRNAs. The results showed that the alternative first exon (AF) events accounted for more than 80% of the total AS events (Fig. 6B), a splicing pattern similar to that observed in humans and mice [55], where AF events also dominate. Figure 6C shows an example AF event of a vlncRNA (HHV-5_lncRNA_548), with read alignments displayed in the IGV browser, providing evidence of the presence of AF in vlncRNAs. Additional IGV views of other representative splicing events are provided in Fig. S8. Retained intron (RI) and Alternative 3′ splice site (A3) both accounted for more than 5% of the total AS events, while the remaining types including Alternative 5′ splice site (A5), Exon skipping (SE), Alternative last exon (AL), and Mutually exclusive exons (MX) each accounted for less than 5% of AS events. Interestingly, the AS patterns of vlncRNAs differed between enveloped and nonenveloped viruses. In enveloped viruses, AF accounts for 91.07%, followed by RI at 3.92%, while in nonenveloped viruses, three AS events with proportions exceeding 10% including AF (40.00%), RI (24.67%), and A3 (17.33%) (Table S6). Comparatively, vlncRNAs were predominantly enriched for AF events (82.45%), whereas vmRNAs displayed a broader distribution, with AF (47.67%), alternative last exon (AL, 17.22%), and RI (13.37%) as the most frequent types. These results suggest that vlncRNAs are more biased toward AF events, while vmRNAs exhibit a more diverse splicing pattern, reflecting distinct regulatory strategies between coding and noncoding viral transcripts.

### Construction of a vlncRNA database

**Figure 7:**
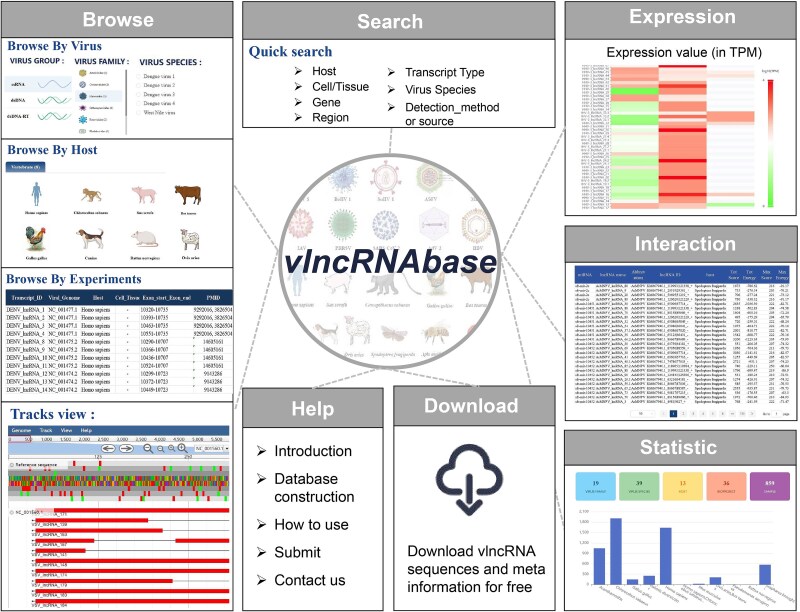
The structure of the vlncRNAbase. It mainly includes pages of browse, search, expression, interaction, statistic, download, and help.

Finally, a database named vlncRNAbase was created to store and organize the vlncRNAs identified above. Besides, 15 vlncRNAs experimentally-validated by PCR-based methods (RT-qPCR, RACE-PCR, etc.) and Sanger sequencing, and 1,089 vlncRNAs that have been identified through high-throughput methods were curated from public databases including NCBI RefSeq, GenBank and RNAcentral, and were also provided in the database. The vlncRNAbase is freely available to the public at http://computationalbiology.cn/vlncRNAbase/#/. It mainly includes pages of Browse, Search, Expression, Interaction, Statistic, Download, and Help which were described briefly as follows (Fig. 7).

*Browse.* This page displays vlncRNAs by virus, host or experiments. When browsing by virus, a virus of interest should be selected, and the vlncRNAs identified for the virus were provided in a table. The details about the vlncRNAs such as strand, abundance, sequence, and detection method or source are provided. These vlncRNAs can also be visualized in a genome browser. When browsing the vlncRNAs by host, a host of interest is first selected; then, viruses infecting the host are provided in a table. The vlncRNAs identified for these viruses can be visualized as above. When browsing the vlncRNAs by experiment, all experimentally-validated vlncRNAs are provided in a table.

*Expression*. This page displays the expression values of vlncRNAs in TGS samples. Firstly, a bioproject is selected; then, the expression values of vlncRNAs identified in the bioproject are shown in a heatmap. The Run accession number, the cell or tissue of the sample, the expression value, and the vlncRNAs ID appear in a small window when hovering over the heatmap.

*Search.* Users can search for vlncRNAs of interest by virus, host, cell or tissue, gene, genomic regions, and detection method or source.

*Interaction*. The page displays the interactions between host miRNAs and vlncRNAs predicted by miRanda (version 3.3a).

*Statistic.* This page displays a summary statistic about the number of vlncRNAs by virus host and the length distribution of vlncRNAs for each virus.

*Download.* The details of vlncRNAs stored in the database are freely available for downloading by virus which is organized by virus family.

*Help.* This page displays the method about the data curation process and the tutorial of using vlncRNAbase.

## Discussion

The vlncRNA has been reported to play important regulatory roles in virus infections [20, 22–24]. Although an increasing number of vlncRNAs have been reported in recent years, most of these studies have focused on one or a few vlncRNAs, or vlncRNAs from one or a few viruses. This study for the first time systematically identified and characterized vlncRNAs from over 20 viral species by mining the viral infection-related TGS data, which provides a valuable resource for further study of vlncRNAs. Additionally, the structure, function, dynamics and biogenesis mechanism of vlncRNAs were further investigated, which deepens our understanding of vlncRNAs.

Previous studies have shown that the ability of different viruses to encode RNA products varies widely, including circular RNAs [56, 57] and small RNAs [53, 58]. This study found that the ability of encoding vlncRNAs also varied much among viruses, with most vlncRNAs identified from dsDNA viruses, followed by ssRNA (+) viruses. Interestingly, viruses of the *Herpesviridae* family were found to encode a large number of vlncRNAs (Fig. 2A), likely due to their large genome size. Although two novel vlncRNAs were experimentally validated in the study, further efforts are needed to verify the existence of vlncRNAs and clarify their functions.

Viral RNAs have been reported to mimic host RNAs to exert their functions [54]. For example, miR-K12-11 of the KSHV can mimic host miR-155 to attenuate transforming growth factor beta (TGF-β) signaling, thus facilitating viral infection and tumorigenesis [54]. Unfortunately, we did not find any reports about the mimicry of host lncRNAs by vlncRNAs. This may be due to the difficulty in identifying sequence similarity between lncRNAs since the lncRNAs lack conserved seed regions as miRNAs. Surprisingly, a large number of vlncRNAs were observed to be structurally similar to hlncRNAs. Among them, vlncRNAs encoded by VACV showed the strongest mimicry of hlncRNAs. Considering that the VACV stably generated vlncRNAs throughout the infection period, vlncRNAs may play an important role in viral infection.

Consistent with their presumed regulatory rather than coding function, vlncRNAs exhibit significantly shorter lengths and lower GC content, which promotes the formation of flexible and less stable secondary structures. These structural characteristics may facilitate transient interactions with host RNA-binding proteins or nucleic acids [59–61], thereby enhancing the structural plasticity necessary for molecular mimicry and functional adaptation during infection. Similar trends have also been reported previously [62], supporting that such differences between vlncRNAs and vmRNAs are expected. However, we note that annotation or detection biases may also contribute and thus biological interpretations should be made with caution.

Computational prediction of vlncRNA function is challenging. This study inferred the function of vlncRNAs from those of structurally-similar hlncRNAs based on the hypothesis that similar structures suggest similar functions, which has been validated and used extensively at the protein level. Although this hypothesis may not hold for lncRNAs, our analysis showed that nearly all human-mimicry vlncRNAs bound to similar miRNAs as their corresponding virus-like hlncRNAs, which support the hypothesis to some extent. Besides, we also observed a weak but statistically significant positive correlation between the expression level of vlncRNAs and hlncRNAs, suggesting they may cooperate in viral infections. In addition, the observed enrichment of vlncRNAs in RISC and RNAi effector complexes is intriguing, raising the possibility that they could modulate viral life cycle stages or contribute to immune evasion, although further mechanistic studies will be required to clarify these roles.

**Figure 8:**
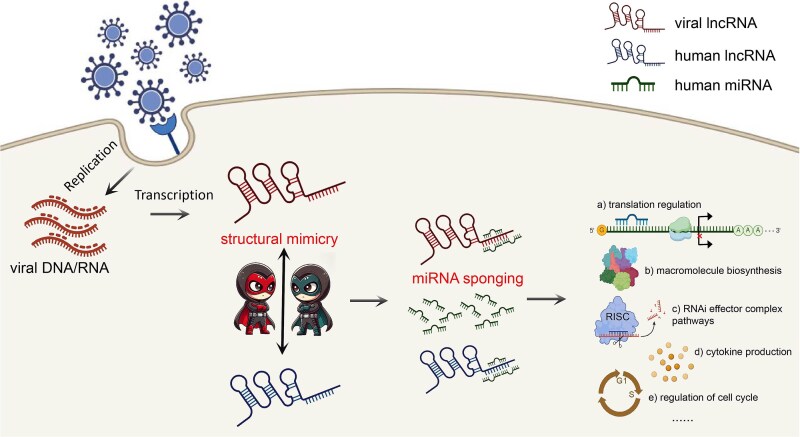
The hypothesized mechanism of how vlncRNAs function in the host cell. The vlncRNA may act as miRNA sponge by mimicking the hlncRNA structures, which cooperates with hlncRNAs to inhibit miRNA function and promote the essential life processes of the host cell, thus facilitating viral proliferation.

The lncRNA plays an important role in translation regulation by binding to miRNAs [8]. Our results showed that virus-like hlncRNAs were mainly involved in the negative regulation of gene silencing, possibly by binding to miRNAs. Interestingly, the human-mimicry vlncRNAs shared a significant number of miRNAs with the corresponding virus-like hlncRNAs and these shared miRNAs were mainly involved in negative regulation of essential life processes. This suggests that the vlncRNA may act as a miRNA sponge, similar to the role of circular RNAs, by mimicking the structure of host lncRNAs. Both the vlncRNA and human lncRNA cooperatively inhibit the function of miRNA and promote the essential life processes of the host cell, which may facilitate the proliferation of the virus (Fig. 8). Notably, vlncRNAs may have therapeutic applications. Therapeutically inhibiting vlncRNAs could restore miRNA function and limit viral proliferation. In addition, RNA-based approaches such as synthetic miRNA mimics may provide feasible strategies to modulate vlncRNA activity.

In addition to the diversity of vlncRNA function, AS and exon usage add another layer of complexity to their regulation and function. For example, alternative first exon usage may alter the TSS of viral lncRNAs, generating distinct isoforms with specific biological effects. In Epstein–Barr virus (EBV), BART lncRNAs (BamHI A rightward transcripts) originate from the BamHI A region and comprise multiple isoforms with alternative first exons, fine-tuning latency–lytic expression and contributing to immune evasion [63]. In Herpes Simplex Virus (HSV), the latency-associated transcript (LAT) exhibits exon variability; notably, its promoter and 5′ exon region displays high levels of histone acetylation during latency, enhancing transcript stability and influencing host chromatin regulation [64, 65]. In HIV-1, noncoding transcripts with variable first exon lengths show differences in splicing efficiency and nuclear retention, which in turn regulate viral latency and reactivation [66–68]. These findings highlight the splicing regulation of vlncRNAs may be a key mechanism of viral adaptation.

This study has several limitations. Firstly, the vlncRNAs identified in the study may include subgenomic RNAs (sgRNAs) from positive-strand RNA viruses such as SARS-CoV-2 because distinguishing between noncoding sgRNAs and lncRNAs is challenging, especially for sgRNAs lacking the leader transcription regulatory sequence. We only identified 1 sgRNA-like vlncRNAs in SARS-CoV-2 (Table S7) based on the characteristic sequence features reported in literature [69]. In fact, noncoding sgRNAs can also be considered as lncRNAs. Secondly, despite the inclusion of over 20 viruses, the list is still small. Besides, the samples used in the study have a bias towards some viruses such as herpesviruses, requiring periodic updates to capture the diverse landscape of novel vlncRNAs in more viruses. Moreover, the extreme dominance of dsDNA viruses (97.94% of vlncRNAs) could partly reflect sampling bias in the 870 TGS datasets. Thirdly, only two vlncRNAs were experimentally-validated due to limited virus resource in our lab. More efforts are needed to extensively validate the vlncRNAs in diverse viruses. Lastly, the functions of vlncRNAs may have been overlooked for a long time. Much more effort is needed to clarify the function and the biogenesis mechanism of vlncRNAs.

## Conclusion

Overall, this study systematically identified and characterized a large number of vlncRNAs from more than 20 virus species for the first time. We found that vlncRNAs may act as a miRNA sponge by structurally mimicking the host lncRNAs. The study deepens our understanding of the diversity and complexity of vlncRNAs and provides a valuable resource for further studies. Besides, it provides new insights into the structure and function of the vlncRNAs.

**Key Points**

- Using third-generation sequencing (TGS), the study identified 5,053 novel virus long noncoding RNAs (vlncRNAs) from 25 viral species, with two validated experimentally via RT-qPCR.
- 772 vlncRNAs from 15 human viruses structurally mimic hlncRNAs despite lacking sequence homology, indicating potential functional mimicry.
- Human-mimicry vlncRNAs bind to similar miRNAs as host lncRNAs, potentially acting as miRNA sponges to regulate host processes.
- A comprehensive database (vlncRNAbase) was developed to store and explore the newly identified and known vlncRNAs, freely available at http://computationalbiology.cn/vlncRNAbase/#/

## Supplementary Material

TableS1_bbaf640

TableS2_bbaf640

TableS3_bbaf640

SupplementS1_S8_TableS4_S7_bbaf640

## Data Availability

All data used in the study are available in supplementary materials. The vlncRNAbase is publicly available at http://computationalbiology.cn/vlncRNAbase/#/. All BAM files containing long reads mapped to viral genomes have been deposited in Zenodo (https://zenodo.org/records/17163336).
