# Supplementary material for: Systematic identification and characterization of virus lncRNAs suggests extensive structural mimicry of host lncRNAs
Source: Brief Bioinform. 2025 Dec 1;26(6):bbaf640. doi: 10.1093/bib/bbaf640 (PMC12667279; doi:10.1093/bib/bbaf640)
Supplement: SupplementS1_S8_TableS4_S7_bbaf640 [file supplements1_s8_tables4_s7_bbaf640.docx]

# Supplementary Materials for

**Systematic identification and characterization of virus lncRNAs suggests extensive structural mimicry of host lncRNAs**

**Figure S1.** Distribution of vlncRNA (A) TPM and (B) length.


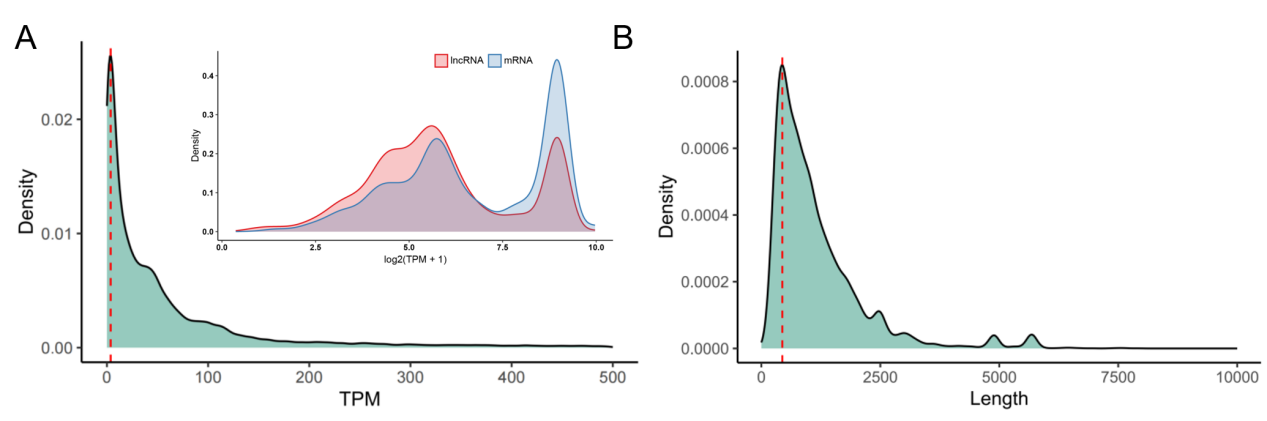


**Figure S2.** Percentage of canonical and novel viral transcripts identified for different virus species. The identified transcripts were classified according to the class_code marked by GffCompare, with “Known transcripts” representing canonical transcripts with class_code of “=”, “c”, “Novel transcripts of known genes” representing novel transcripts of known genes with class_code of “j”, “k”, “m”, “n”, and “Novel transcripts of novel genes” represents novel transcripts from new genes with class_code of “i”, “x”, “u”, “y”, “o”, “Others” represent RNA fragments or artifacts due to experimental or sequencing techniques with class_codes of “p”, “e”, “s”, “r”. The bar plot above shows the total number of viral transcripts produced by each viral species.

**
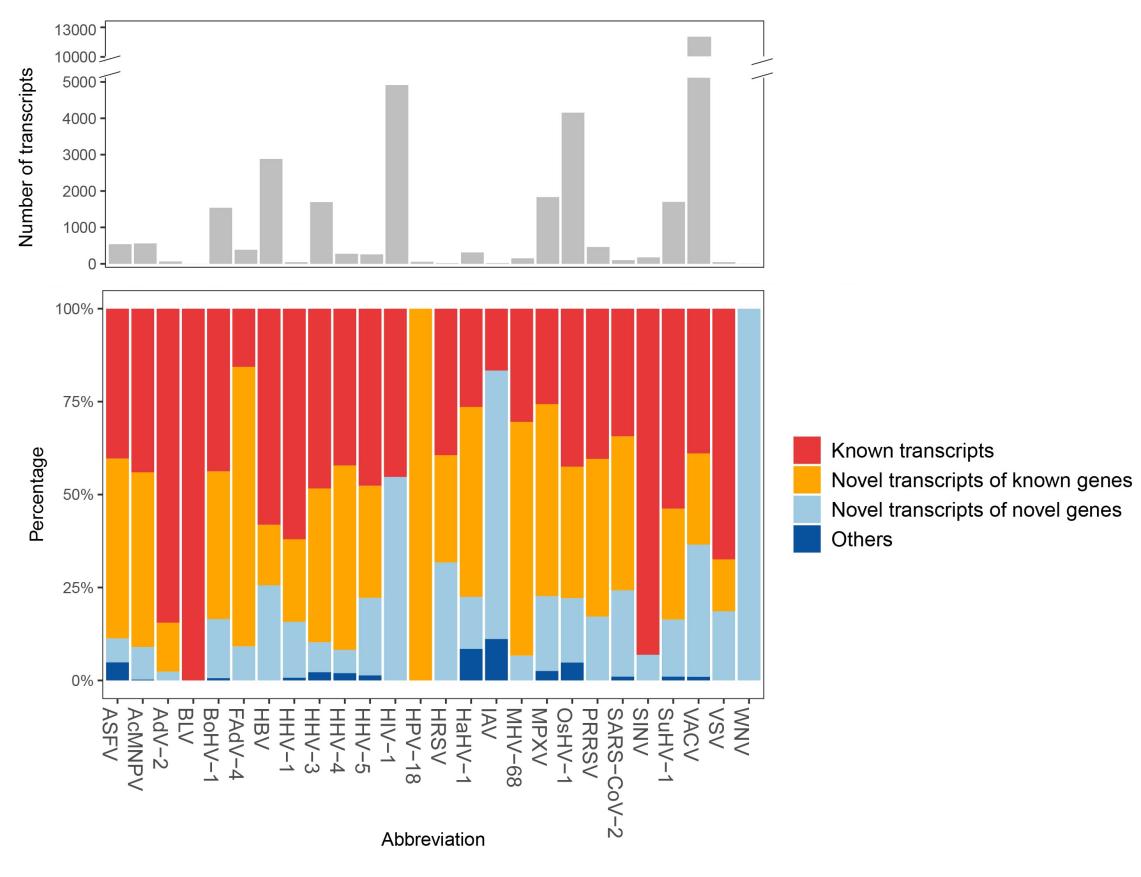
**

**Figure S3.** RT-qPCR validation of vlncRNA expression.


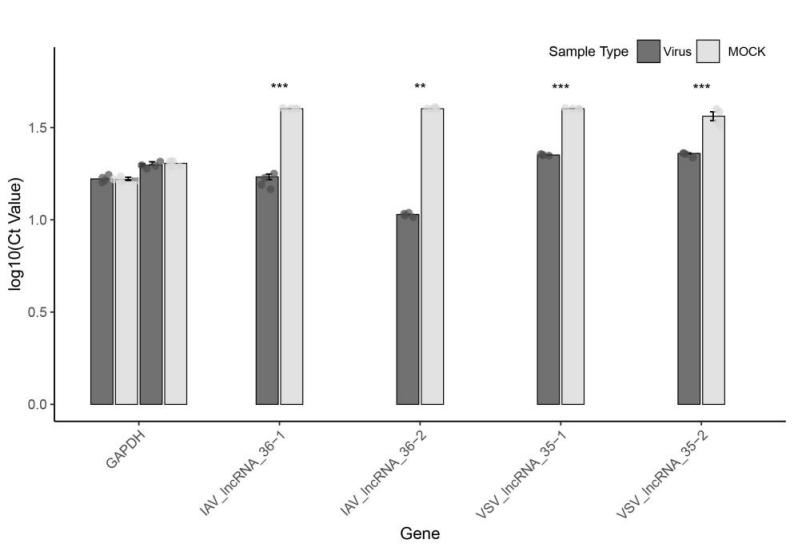


**Figure S4.** The number of vmRNAs that were classified into four RNA types based on the Rfam.


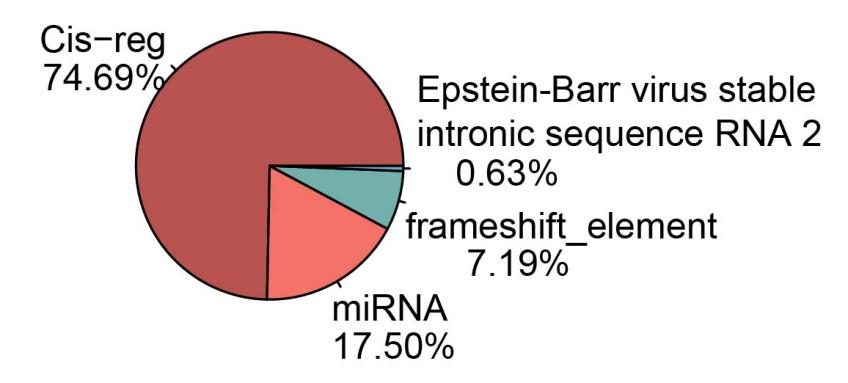


**Figure S5.** (A)~(B) The enriched cellular component and KEGG pathways for human miRNAs that interact simultaneously with virus-like hlncRNAs and human-mimicry vlncRNAs.

**
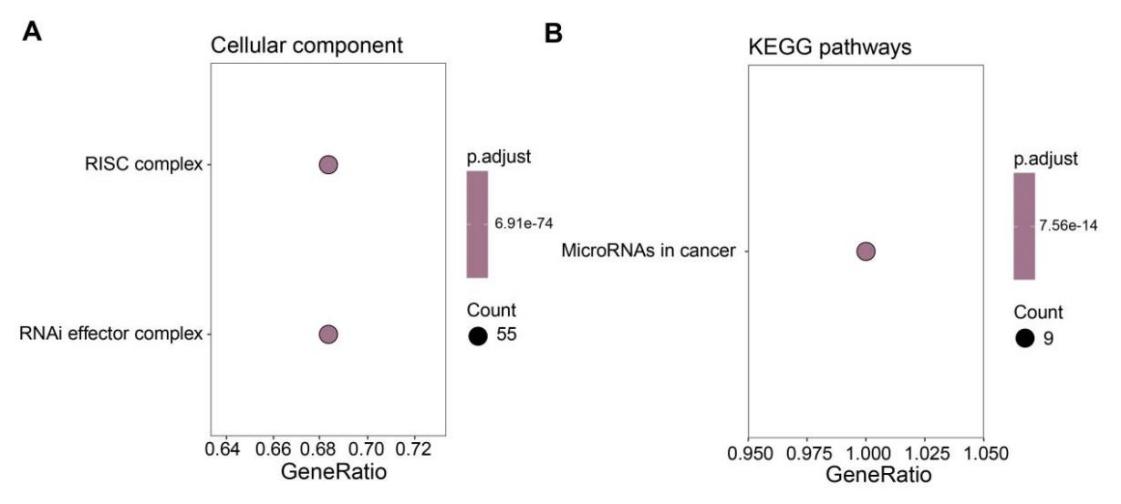
**

**Figure S6.** (A) The expression correlation between human-mimicry vlncRNAs and virus-like hlncRNAs. (B) The expression correlation between human-mimicry vlncRNAs and randomly selected non-mimicry virus-like hlncRNAs.

**
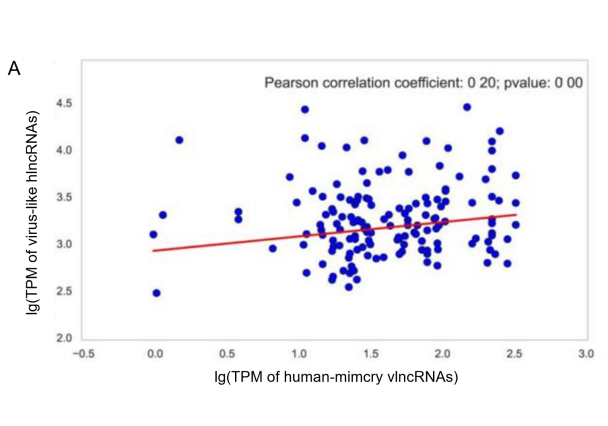

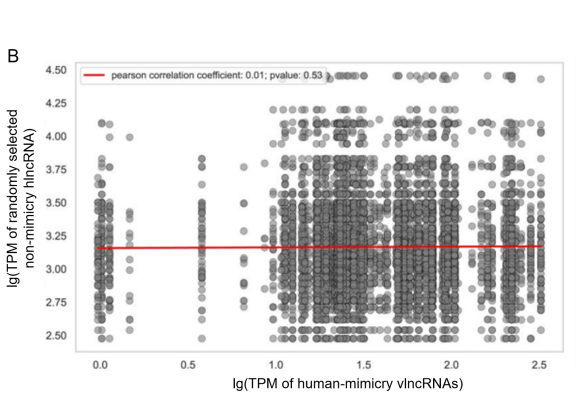
**

**Figure S7.** The number of per sample novel vlncRNAs detected at different hours post-infection (hpi) of Autographa californica multiple nucleopolyhedrovirus (AcMNPV), Adenovirus type 2 (AdV-2), Influenza A Virus (IAV), Ostreid herpesvirus 1 (OsHV-1), Indiana vesiculovirus (VSV), West Nile virus (WNV), and Severe acute respiratory syndrome-related coronavirus (SARS-CoV-2).

**
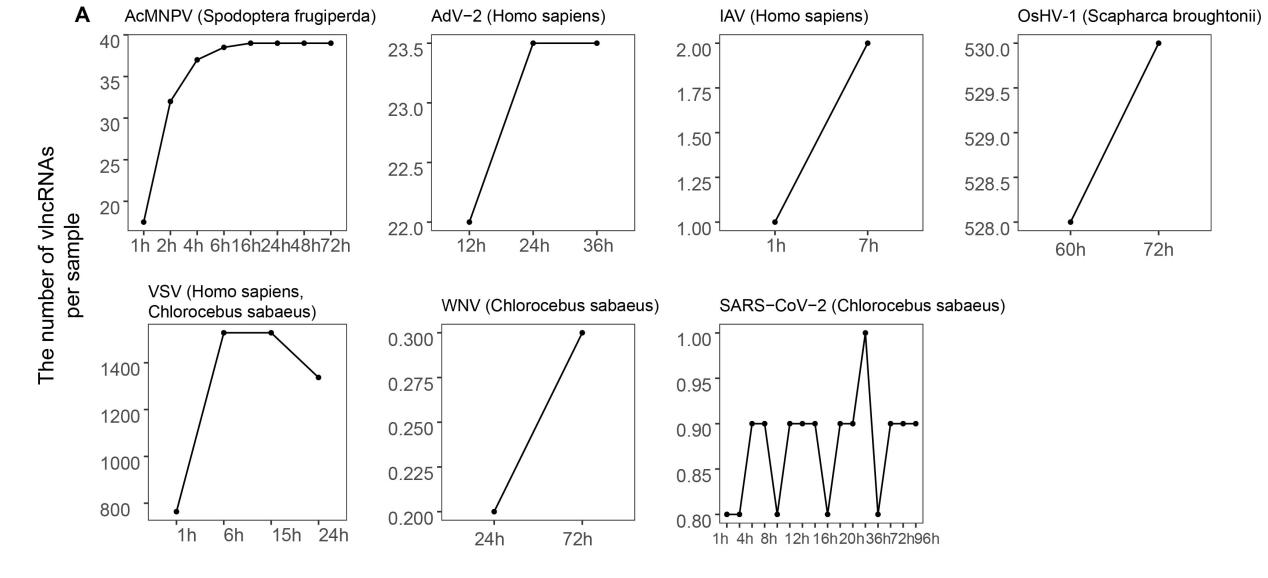
**

**Figure S8.** Example of Retained intron (RI), Alternative 3’ splice site (A3), Alternative 5’ splice site (A5), Exon skipping (SE), Alternative last exon (AL) and Mutually exclusive exons (MX) events. Read alignments were visualized in the Integrative Genomics Viewer (IGV) using BAM files mapped to the corresponding viral genomes, with zoomed-in views highlighting the alternative splicing regions.

**
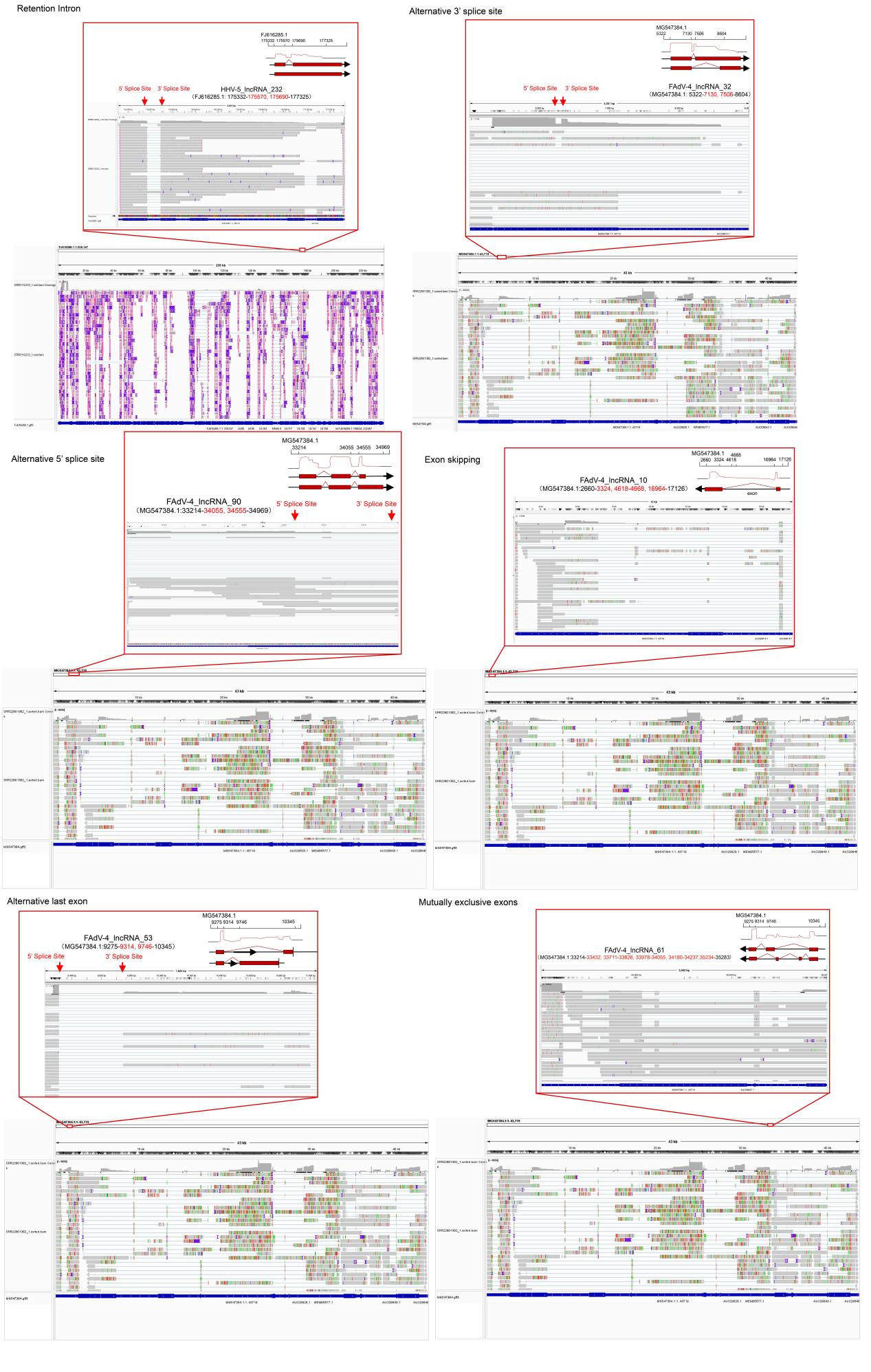
**

**Table S4.** The primers used for RT-PCR.

| **Primer designation** | **Primer sequence** | **Target region** | **Product size** |
| --- | --- | --- | --- |
| GAPDH(human) (F) | 5′-TTTTGCGTCGCCAGCC-3′ | 40-55 | 208bp |
| GAPDH(human) (R) | 5′-ATGGAATTTGCCATGGGTGGA-3′ | 247-227 |  |
| GAPDH(dog) (F) | 5′-GTAGTGAAGCAGGCATCGGA-3′ | 837-856 | 108bp |
| GAPDH(dog) (R) | 5′-GTCGAAGGTGGAAGAGTGGG-3′ | 944-925 |  |
| VSV-lncRNA-35-1 (F) | 5′-TGGAAGAGCATAAGAATCTGAACCA-3′ | 146-164 | 161bp |
| VSV-lncRNA-35-1(R) | 5′-CAGGTGGTTTCCAGTCCGAA-3′ | 296-315 |  |
| VSV-lncRNA-35-2 (F) | 5′-GGATCAGGCGGTAGGAGAGA-3′ | 63-82 | 108bp |
| VSV-lncRNA-35-2(R) | 5′-TGGTTCAGATTCTTATGCTCTTCCA-3′ | 146-179 |  |
| IAV-lncRNA-36-1 (F) | 5′-GCCTAGCAAACAATGGCGAA-3′ | 362-381 | 187bp |
| IAV-lncRNA-36-1(R) | 5′-GTGCCGCAGTAGCAAGTGG-3′ | 530-548 |  |
| IAV-lncRNA-36-2(F) | 5′-TGGCTTTCCTGAATCCCTTTGT-3′ | 141-162 | 74bp |
| IAV-lncRNA-36-2(R) | 5′-CCCCTTTGGCTTGTGTTGAG-3′ | 309-290 |  |

**Table S5.** Full names of viruses that had novel vlncRNAs identified in the study.

| **Viruses** | **Abbreviation** |
| --- | --- |
| Alphapapillomavirus 7 | HPV-18 |
| Human Respiratory Syncytial Virus | HRSV |
| Human betaherpesvirus 5 | HHV-5 |
| Human mastadenovirus C | AdV-2 |
| Bovine alphaherpesvirus 1 | BoHV-1 |
| Human immunodeficiency virus 1 | HIV-1 |
| Human gammaherpesvirus 4 | HHV-4 |
| Suid alphaherpesvirus 1 | SuHV-1 |
| Autographa californica multiple nucleopolyhedrovirus | AcMNPV |
| Porcine reproductive and respiratory syndrome virus | PRRSV |
| Fowl aviadenovirus C | FAdV-4 |
| African swine fever virus | ASFV |
| Severe acute respiratory syndrome-related coronavirus | SARS-CoV-2 |
| Haliotid herpesvirus 1 | HaHV-1 |
| Ostreid herpesvirus 1 | OsHV-1 |
| Human alphaherpesvirus 3 | HHV-3 |
| Sindbis virus | SINV |
| Indiana vesiculovirus | VSV |
| West Nile virus | WNV |
| Human alphaherpesvirus 1 | HHV-1 |
| Influenza A virus | IAV |
| Hepatitis B virus | HBV |
| Monkeypox virus | MPXV |
| Murine herpesvirus 68 | MHV-68 |
| Vaccinia virus | VACV |

**Table S6.** The alternative splicing (AS) patterns of vlncRNAs in enveloped and non-enveloped viruses.

| Alternative splicing type | Enveloped (n=739) | Non-enveloped (n=150) | Total |
| --- | --- | --- | --- |
| Alternative first exon (AF) | 673 (91.07%) | 60 (40.00%) | 733 |
| Alternative 5' splice site (A5) | 29 (3.92%) | 4 (2.67%) | 33 |
| Alternative 3' splice site (A3) | 19 (2.57%) | 26 (17.33%) | 45 |
| Retained intron (RI) | 13 (1.76%) | 37 (24.67%) | 50 |
| Skipped exon (SE) | 3 (0.41%) | 12 (8.00%) | 15 |
| Alternative last exon (AL) | 2 (0.27%) | 10 (6.67%) | 12 |
| Mutually exclusive exon (MX) | 0 (0.00%) | 1 (0.67%) | 1 |
| Total | 739 | 150 | 889 |

**Table S7.** sgRNA candidates identified in SARS-CoV-2. Candidate sgRNAs were defined as vlncRNAs containing a ~70-nt leader sequence at the 5’ end with a TRS-L motif (ACGAAC, allowing ≤1 mismatch), and at least one body TRS (TRS-B) motif (ACGAAC, ACGGAC, or ACAAAC) located upstream of ORF, according to Wong’s study^[1]^.

| Virus | ViralGroup | Total lncRNA | The number of sgRNA | The candidate sgRNAs | The ratio of sgRNA |
| --- | --- | --- | --- | --- | --- |
| SARS-CoV-2 | ssRNA(+) | 14 | 1 | SARS-CoV-2_lncRNA_2 | 7.14% |

[1] Wong, C.H., Ngan, C.Y., Goldfeder, R.L., Idol, J., Kuhlberg, C., Maurya, R., Kelly, K., Omerza, G., Renzette, N., De Abreu, F. et al. (2021) Reduced subgenomic RNA expression is a molecular indicator of asymptomatic SARS-CoV-2 infection. Communications Medicine, 1, 33.
